# East Asian Monsoon Signals Reflected in Temperature and Precipitation Changes over the Past 300 Years in the Middle and Lower Reaches of the Yangtze River

**DOI:** 10.1371/journal.pone.0131159

**Published:** 2015-06-24

**Authors:** Zhixin Hao, Di Sun, Jingyun Zheng

**Affiliations:** 1 Institute of Geographic Sciences and Natural Resources Research, Chinese Academy of Sciences, Beijing, China; 2 University of Chinese Academy of Sciences, Beijing, China; Institute of Tibetan Plateau Research, CHINA

## Abstract

Based on observational data and Asian monsoon intensity datasets from China, the relationships between the East Asian winter monsoon index and winter temperature, the East Asian summer monsoon index and Meiyu precipitation over the middle and lower reaches of the Yangtze River, were analyzed. We found that the monsoon signals were reflected in the temperature and Meiyu precipitation variations. Thus, we used the reconstructed Meiyu precipitation and winter temperature series for the past 300 years and detected the summer/winter monsoon intensity signals using multi-taper spectral estimation method and wavelet analysis. The main periodicities of Meiyu precipitation and winter temperature, such as interannual cycle with 2–7-year, interdecadal-centennial cycles with 30–40-year and 50–100-year, were found. The good relationships between the East Asian summer and winter monsoons suggested that they were in phase at 31-year cycle, while out of phase at 100-year cycle, but with 20-year phase difference. In addition, the winter monsoon intensity may be regulated by the North Atlantic Oscillation, the Arctic Oscillation and the Atlantic Multidecadal Oscillation, and the summer monsoon is closely related to the signal intensities of the Pacific Decadal Oscillation.

## Introduction

The East Asian monsoon is one of the most important atmospheric circulation systems from middle to high latitudes in the Northern Hemisphere [1]. Abnormal East Asian monsoon has important influence on temperature and precipitation over eastern China, Japan, South Korea, and neighboring countries and regions; extreme monsoon years may even influence national economies [2–5]. Climatic disaster events related to monsoon anomalies have accelerated the pace of research over the last 10 years, in order to improve our understanding of the causes of variations on the East Asian monsoon, and to explore the relationships with atmospheric circulation systems. These topics have become a hot topic in the climate research field, especially in relation to the establishment of disaster early-warning systems, which require dynamic real-time predictions to mitigate economic losses.

So far, the studies have been limited by the paucity of historical observational data, as most indices representing East Asian winter monsoon (EAWM) and East Asian summer monsoon (EASM) intensities were established after the 1950s; thus, the indices can only provide data for climate variations at interannual time scales, and most series are too short to examine monsoon intensity changes at interdecadal–centennial time scales.

In 2007, Yancheva et al. [6] and Zhang and Lu [7] disputed whether the collapse of the Tang Dynasty was associated with the development of an anti-phase relationship between the EAWM and EASM; since then, the development of long-term high-resolution reconstructions of intensity changes of the EAWM and EASM has become an urgent priority. However, the most related studies have focused on long-term paleo-monsoon variations since the latest quaternary at orbital–millennial–centennial time scales [8–10]. Recently, researchers have investigated their relationship using historical evidences obtained from stalagmites, lake sediments, ice cores, loess and other paleoclimate proxy data [11], and the results showed that, at orbital scales, the EAWM and EASM were out of phase during the last glacial maximum, but were in phase during the Holocene. However, at millennial time scales, the relationship was so complex that the research results were not consistent. Some researchers have indicated that the anti-phase relationship between the monsoons mainly occurred in northern China during the early-middle Holocene, and that the correlation between the two was not significant until the late Holocene [12]. Few studies have analyzed the monsoon intensity derived from high-resolution proxy records over recent hundreds of years at interannual–interdecadal time scales. In addition, most high-resolution series related to monsoon intensity changes represented reconstructions of EASM [9, 13–18], and the reconstruction of Zhu et al. [19] showed possible intensity changes of the EAWM on the basis of winter temperature series derived from tree rings at Changbai Mountain, northeast China.

Over East Asia, the EASM and EAWM are two important aspects of the East Asian monsoon system due to seasonal transition features. During summer time, the rainfall intensity and variations are closely related to the fluctuations of the summer monsoon. One of the major rain belts—Meiyu—generally stays over the middle and lower reaches of the Yangtze River (MLRYR) during June and July. For years with strong summer monsoons, less Meiyu precipitation and more drought events have occurred in this region, and vice versa [2, 20–21]. During winter time, the temperature over eastern China is closely related to the winter monsoon intensity. The MLRYR located at the southern boundary is influenced by the EAWM, and the annual variation of temperature can be used as an indicator for the intensity of the EAWM. In addition, most of the various EAWM intensity indices derived from the different methods have significant correlations with temperature over this region [19, 22]. Thus, it is possible that the reconstructed Meiyu precipitation and temperature series over the MLRYR contain EASM and EAWM signals.

In this paper, we will use the reconstructed Meiyu precipitation and winter temperature in the MLRYR over the past 300 years as a basis for detecting signals of the EASM and EAWM at interannual—interdecadal—centennial time scales, and for analyzing possible relationships with the climatic modes, including Pacific Decadal Oscillation (PDO), the North Atlantic Oscillation (NAO), the Atlantic Multidecadal Oscillation (AMO) and the Arctic Oscillation (AO). The results will contribute to improvement of the ability of model simulations to predict the characteristics and frequencies of monsoon and climate-related disaster events.

## Data and Methods

### Data

Three types of datasets were used in this study: reconstructed Meiyu precipitation and winter temperature data, published eastern Asian monsoon intensity index, and modern observation data.

#### Reconstructed Meiyu precipitation and temperature

Two reconstruction series of winter temperature [22] and Meiyu precipitation [23] over the MLRYR since 1736 were used, which were both derived from Chinese historical document *Yu* (rainfall)-*Xue* (snowfall)-*Fen* (length unit, 0.32 cm)-*Cun* (3.2 cm). Winter temperature was reconstructed using annual information on the winter snowfall days over the MLRYR region during the period 1736–2000. Meiyu precipitation with annual time resolution was reconstructed based on the length of the monsoon rainy season over this region from 1736 to 2000. The original records in the historical documents were independent from each other in the two series. They were mainly used to detect the annual-centennial winter/summer monsoon signals (Please see S1 Table for downloading original data).

#### Previously published East Asian monsoon indices

Due to the complexity of the East Asian monsoon system, as well as the different research goals, definitions of East Asian monsoon intensity were different among previous studies [24–27]. After analyzing the available EAWM and EASM index series, the EAWM index established by Guo [24] was selected, which mainly reflected the atmospheric circulation situations which have direct influence to winter monsoon intensity at its birthplace. The movement of the winter monsoon over China was mainly controlled by the intensity of the Siberian high, and the weak (strong) winter monsoon generally corresponded to the smaller (larger) extent of the Siberian high [28]. Meanwhile, Guo’s winter monsoon index had a good relationship with winter temperature over the MLRYR [24]. In addition, the EASM index of Zhang et al. [25] not only reflected the interannual intensity of EASM circulation, but also indicated the interannual variations of precipitation and rain belt over eastern China; the weak (strong) summer monsoon usually corresponded to the abundant (short) monsoonal rainfall over the MLRYR, which had a specified synoptic significance and was easy to calculate. We then calculated the spatial correlations between the EAWM index and winter temperature, and between the EASM index and Meiyu precipitation. The winter monsoon index of Guo [24] was used to determine the mean values of the index at three grid points near the Siberian high (60°N, 100°E; 60°N, 90°E; 50°N, 100°E) from the National Center for Atmospheric Research/National Centers for Environmental Prediction (NCAR/NCEP) sea-level pressure dataset for the period 1951–2000. The summer monsoon index was calculated as the June–August mean zonal wind difference between the East Asian tropical monsoon trough region (10–20°N, 100–150°E) and the East Asian subtropical region (25–35°N, 100–150°E) at 850 hPa, based on NCAR/NCEP reanalysis data for the period 1948–1998.

#### Monthly temperature and precipitation data from 1961 to 2000

**Fig 1:**
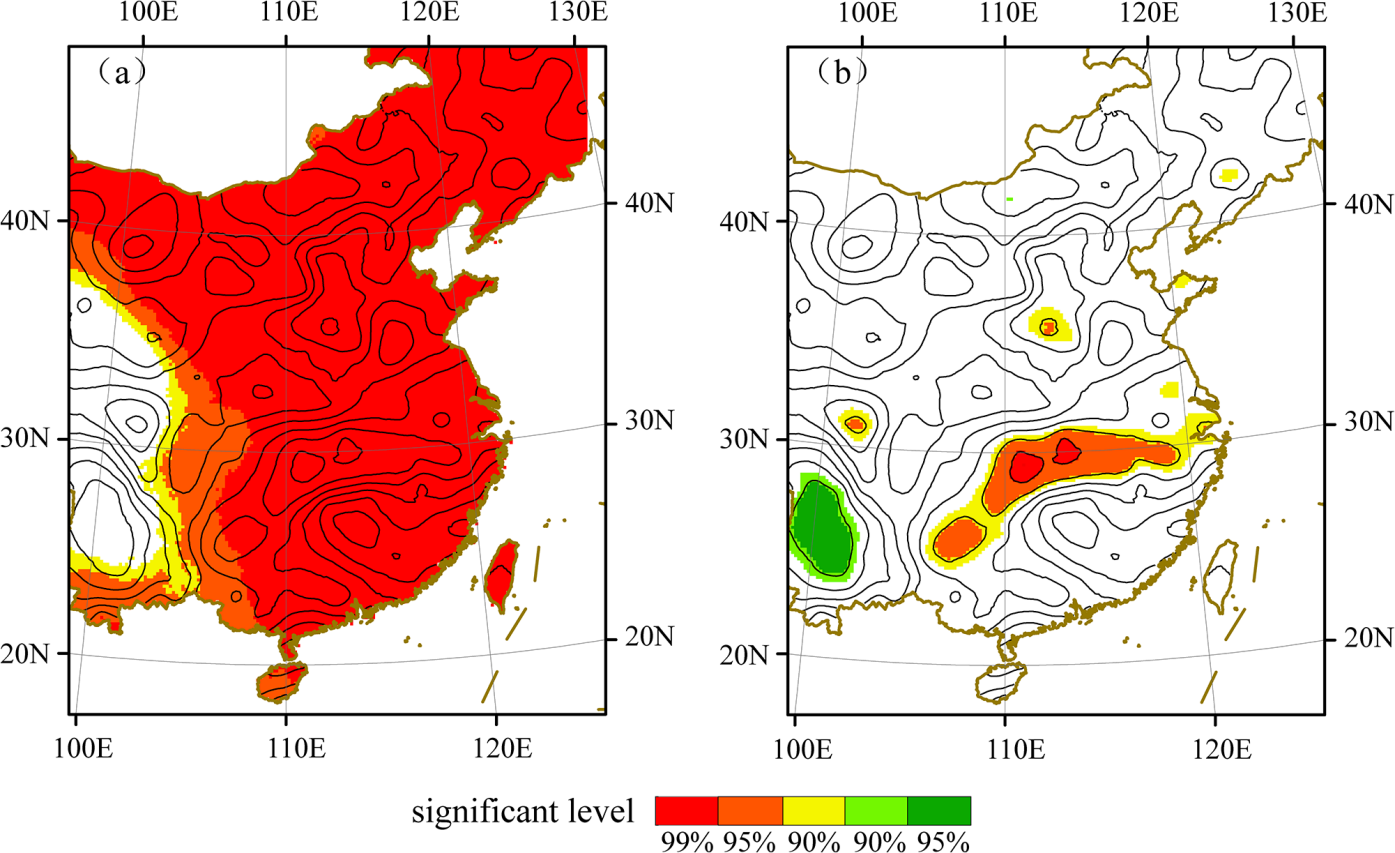
Correlations between East Asian monsoon indices and temperature/precipitation during the observed period. (a) winter monsoon index and winter temperature and (b) summer monsoon index and June–July precipitation. The shaded areas from red to yellow passed negative correlation at 99%, 95%, 90% confidence levels, and green color indicates positive correlated regions.

The observational data were downloaded from the China Meteorological Data Sharing System (http://www.cma.gov.cn//2011qxfw/2011qsjgx/). We calculated December–February temperature anomalies and June–July (Meiyu over MLRYR) precipitation anomaly percentages for 658 stations during the period 1961–2000. The correlation coefficients between the winter (summer) monsoon indices and temperature (precipitation) for the period 1961–2000 are presented in Fig 1. Fig 1 shows that the correlation coefficients between the winter temperature and the winter monsoon index range from -0.5 to -0.7, and from -0.3 to -0.5 between June–July precipitation (main stage of Meiyu) and the summer monsoon index over the MLRYR, passing 90% confidence level. That is, precipitation decreased (increased) during strengthened (weakened) EASM, and winter temperatures decreased (increased) during strengthened (weakened) EAWM, throughout the MLRYR.

#### Climatic modes

In addition, we used reconstructions of the PDO, NAO, AMO and AO to examine the statistical relationships between the temperature/precipitation, winter/summer monsoons and climatic modes. Since the published PDO, NAO, AMO and AO reconstruction series from multi-proxies were not completely consistent between the variations, in order to extract the main common variation features, we downloaded data from the National Oceanic and Atmospheric Administration (NOAA) National Data Center (NCDC) webpage (http://www.ncdc.noaa.gov/data-access/paleoclimatology-data/datasets). The downloaded PDO index included four series from tree-ring proxy [29–32], and one series from Chinese historical documents [33]. The five NAO datasets used included one series from a single tree-ring proxy [34], three series from speleothems, ice cores and tree-ring integrated evidences [35–37], and one series from a European documentary proxy [38]. After collecting reconstructed PDO and NAO data, a principal components analysis method was used to extract their main characteristics, and the first two principal components, both accounting for 53.0% variances of the reconstructed five individual proxy data, were selected to form new PDO and NAO indices for the past 300 years.

As very few AMO and AO indices have been reconstructed dating back 300 years, we only found one AMO [39] and one AO [40] reconstruction with annual resolution in the NCDC open access dataset, both of which were derived from tree-ring proxy. The detected periodicity showed that the AMO has 40–128-year low-frequency oscillation, and the AO has a 51–100-year cycle.

### Analysis method

**Fig 2:**
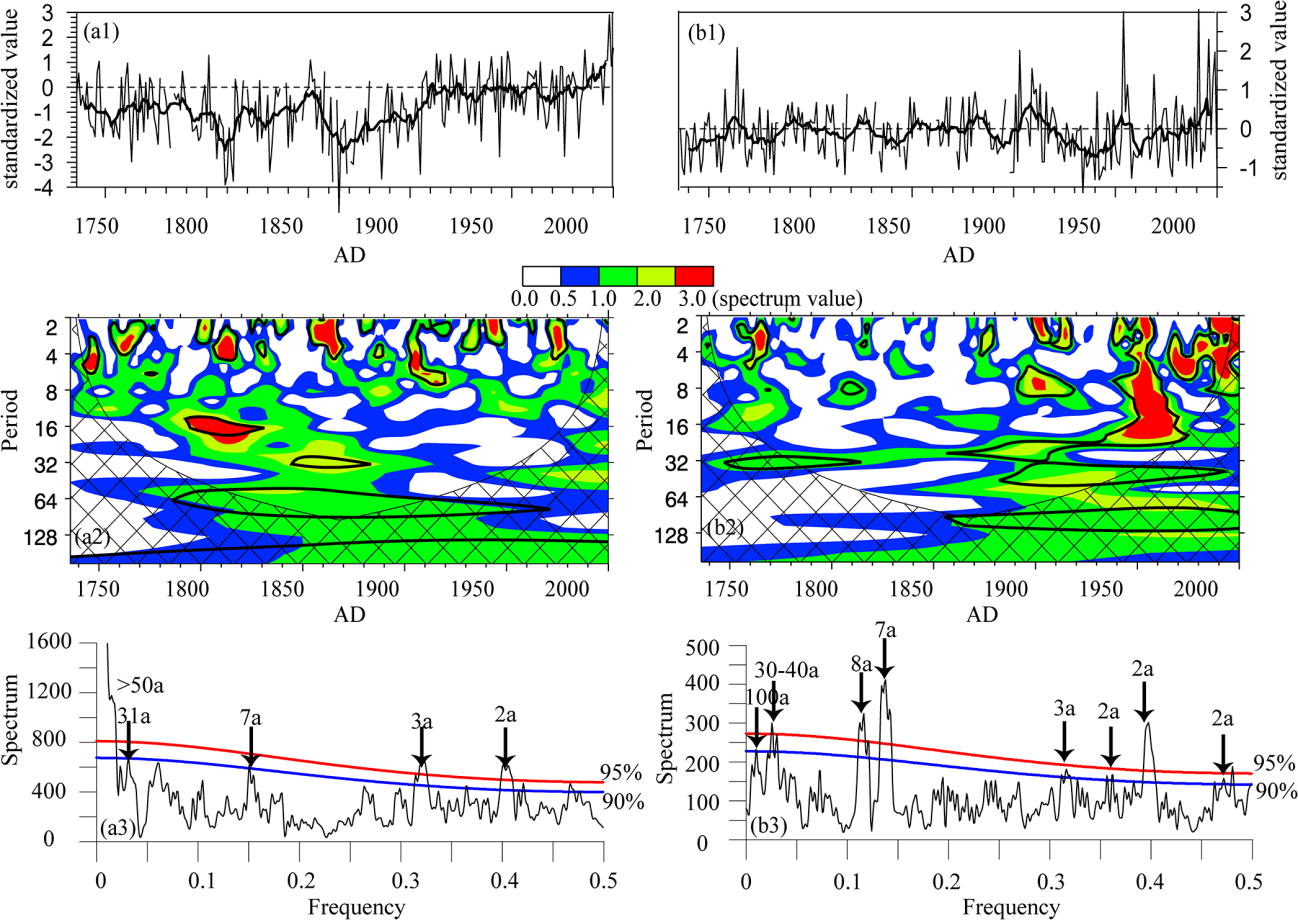
Standardized climate reconstructions and spectra analysis. (a) winter temperature and (b) Meiyu precipitation over the MLRYR. (1) Standardized climate reconstructions; (2) wavelet spectra analysis; region circled by black contours passing the 90% confidence level; cross-hatched areas represent the cone of influence calculated by the standard program from http://ion.researchsystems.com/ [41]; (3) multi-taper method analysis with 90% and 95% confidence levels [42].

To detect the contained East Asian winter and summer monsoons intensity signals from reconstructed temperature and precipitation series at different time scales, we used periodicity analysis with a wavelet statistical tool and a multi-taper spectral estimation method to examine winter temperature and Meiyu precipitation data from 1736 to 2000 [41–42]. The cycle detection results both showed that winter temperature has significant interannual (e.g., 2–7-year) and multidecadal (50–100-year) signals, and Meiyu precipitation has significant 2–7-year, 30–40-year and 100-year signals at the 90% significance level (Fig 2). The wavelet coefficients at corresponding time scales were then extracted to analyze the interannual and multidecadal variations of the two climatic factors.

## Results and Discussion

### Main periodic signal and features of Meiyu precipitation and winter temperature

The observed temperature and June–July precipitation have high correlations with the winter and summer monsoons, respectively, as shown in Fig 1. Thus, the reconstructed temperature and Meiyu precipitation series reflected the intensity signals of the EAWM and EASM for the past 300 years. The 2–7-year cycle existed in the EAWM and EASM intensity index series, and some studies have discussed these interannual variations and their relationships with climatic modes including the El Niño Southern Oscillation and the Tropospheric Biennial Oscillation [43–46]. Thus, here we only focus on the longer time-scale cycles, which were not covered by the current observation data from 1951. For temperature variations (negative relationship with winter monsoon), the multidecadal signals for the 31-year period and the 50–100-year period are shown in Fig 2A, and for Meiyu precipitation variations (negative relationship with summer monsoon), the interdecadal signals for the 30–40-year period and the centennial signal for the 100-year period are shown in Fig 2B. As the Meiyu precipitation and winter temperature over the MLRYR can reflect the EASM and EAWM signals, in the following text, Meiyu precipitation and temperature are replaced by negative EASM and EAWM, respectively.

**Fig 3:**
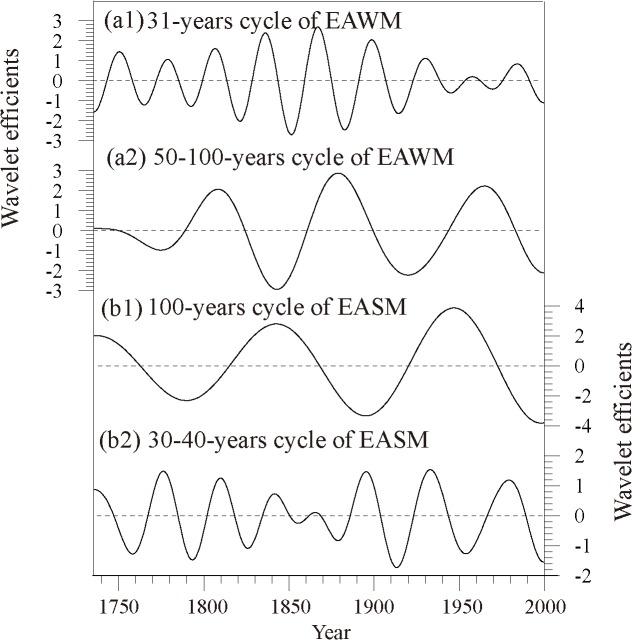
Variations of EAWM and EASM over the interdecadal–centennial scale: (a) EAWM and (b) EASM.

For the variation of winter monsoon (Fig 3A), the 31-year oscillation was very strong for most of the past 300 years. However, during the period 1940–1980, the 2–7-year oscillation was dominant, based on the wavelet analysis. This result was supported by the research work of Pei and Li [47], who developed the EAWM indices from 1899 to 1998, based on the sea-level pressure data of the UK Met Office Hadley Centre, and also found that the 30-year oscillation was one of the significant cycles of the EAWM indices. For the 50–100-year variation of winter monsoon, the intensity was strong during the periods 1736–1790, 1825–1860, 1900–1945 and after 1980, but the intensity from 1736 to 1790 was not as strong as the other three periods. During the periods 1791–1825, 1861–1900 and 1946–1980, the EAWM was weak. Ding et al. [48] found that the EAWM had strong—weak—strong interdecadal variations, i.e., a strong stage from 1950 to 1986/1987, a weak stage from 1986/1987 to 2005, and a strong stage again from 2005, corresponding to the warming hiatus, which also showed an approximate 50–60-year cycle [49]. The EAWM cycles, represented by the winter temperature reconstructions of Zhu et al. [19] at Changbai Mountain in northeastern China, were consistent with our results during the periods 1776–1847 and 1921–2000.

For the variation of EASM (Fig 3B), except for the 1835–1880 period, the 30–40-year oscillation was dominant over the past 300 years. In terms of stage variability, the summer monsoon was strong during the periods 1736–1750, 1770–1785, 1800–1819, 1835–1848, 1860–1870, 1889–1905, 1925–1945 and 1969–1990, but weak during other periods. Similar periodicity in the other EASM index series from the observation and proxy data were also detected, and 30–40-year cycles were the most important modes for EASM [48, 50–51]. Chu et al [52] investigated stalagmite records with annual—decadal resolutions since the medieval warm period, and found that a 42-year periodicity may relate to ocean oscillations. At a 100-year time scale, the EASM was strong in the three periods of 1736–1765, 1821–1870 and 1921–1975, and weak in the other periods of 1766–1820, 1871–1920 and after 1976, which is consistent with the fact that the EASM has weakened since the middle of the 1970s [20]. In addition, the tree-ring based precipitation series related to the EASM from the previous August through to July [53] showed similar variations, i.e., a weak summer monsoon during 1779–1806 and a strong summer monsoon during 1821–1852. The layer thickness of the stalagmite over the middle reaches of the Yangtze River reflected the EASM intensity [54], the periods including the weak summer monsoon in 1880–1920 and the strong summer monsoon in 1921–1960 were consistent with the results from our historical documents. Observations and modeling simulations of the EASM intensities also showed that monsoon circulation was weaker from the late 1890s to the early 1910s, but that summer monsoon circulation was stronger from the 1940s to the 1970s [15], which is in agreement with our findings at centennial time scales.

**Fig 4:**
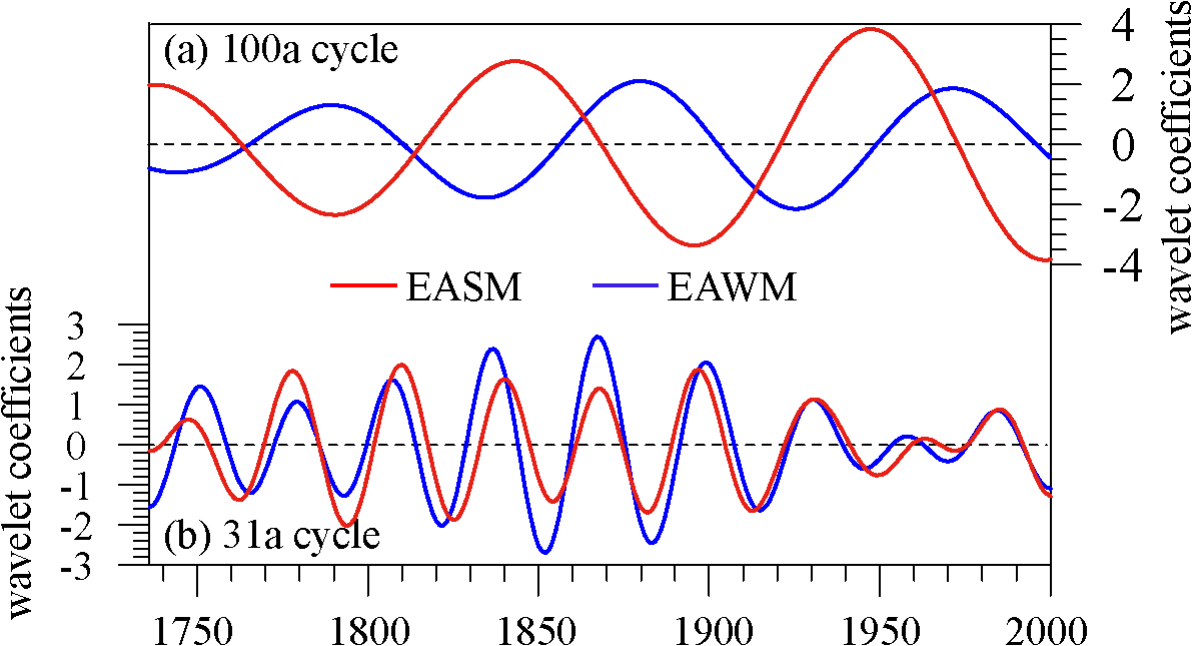
Phase relationship between EAWM and EASM at 100-year (a) and 31-year (b) time scales.

Meanwhile, in order to analyze the phase relationship between EAWM and EASM, the cycles of 100-year and 31-year were extracted (Fig 4). The comparison shows that the EASM and EAWM have a positive correlation at a 31-year time scale, with a coefficient of 0.78 (passing the α = 0.05 significance level), indicating that they are in phase. However, EASM and EAWM have a negative correlation at the 100-year time scale, with a coefficient of 0.50 (based on Bretherton et al. [55], the statistical significance of the correlation between smoothed series has to consider the reduction of the degree of autocorrelation, so the coefficient did not pass the statistical test), which only indicated that they were out of phase. For the 100-year cycle, before 1815, the EAWM and EASM were completely out of phase, but after that, the onset of weakening (strengthening) winter monsoon occurred about 20 years before the strengthening (weakening) of the summer monsoon.

### Climatic modes and EAWM/EASM

**Fig 5:**
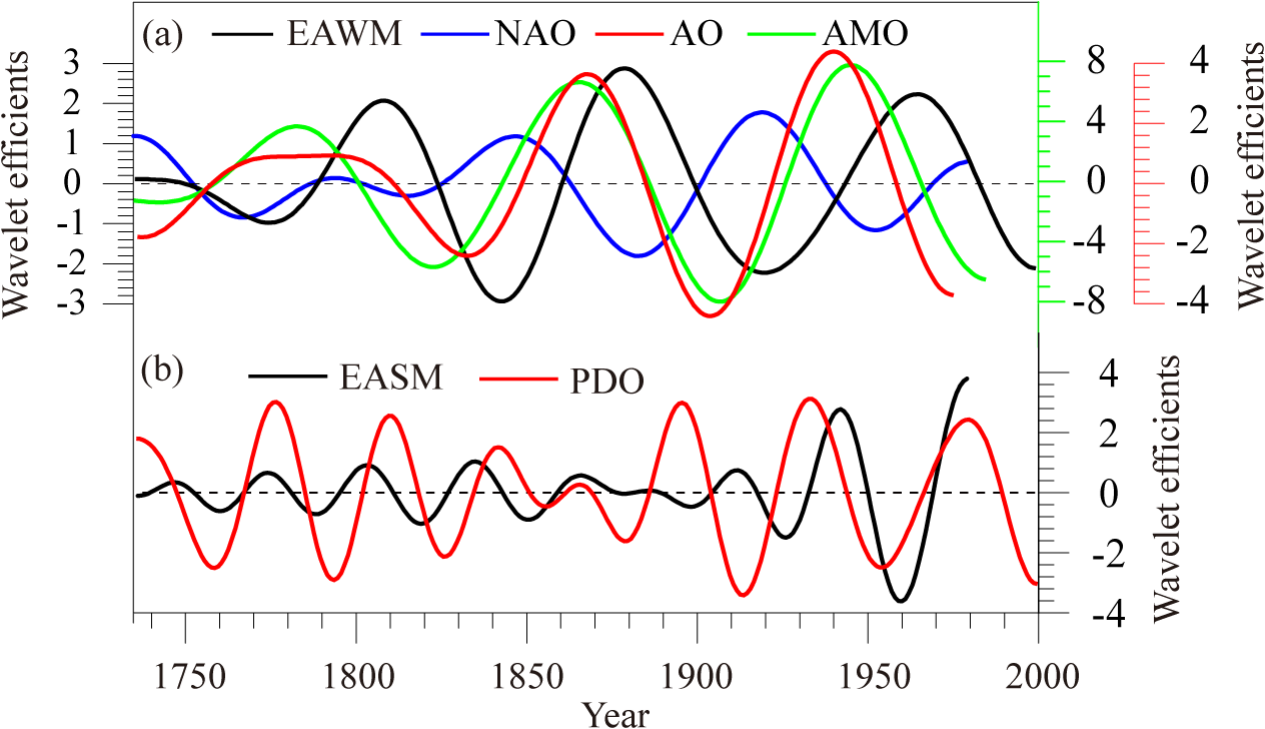
Relationship between EAWM, NAO, AO and AMO at 50–100-year cycles, AO and AMO use the green and red color axes (a), and EASM and PDO at 30–40-year cycles (b).

The causes of long-term variations of the EAWM and EASM are complex, and may vary depending on the time scale investigated. In this study, we detected two main periodic signals associated with long-term changes in the EASM and EAWM, one with a period of 30–40 years, which is consistent with the main mode of the PDO, and the other with a period of 51–100 years, which is consistent with the mode of the NAO, AO and AMO [39–40, 56–58]. Using statistical approaches, we have primarily attempted to identify possible links between winter/summer monsoonal intensities and ocean/atmosphere modes. The extracted wavelet coefficients at 30–40-year time scales for EASM and PDO are shown in Fig 5. The positively correlated coefficient is 0.30 between EASM and PDO. The PDO signal was strong for most of the period 1736–1979, and was interrupted only by a short-term weak signal during 1835–1900; corresponding to this intensity variation, EASM also showed a similar weak signal during the period 1835–1880, but there was an enhanced signal earlier than that of the PDO. At 51–100-year scale, the correlation coefficient between the NAO and the winter monsoon was -0.73. The signal intensity of the NAO can be divided into two stages. The signal was weak prior to 1820, when the winter monsoon was positively related to the changes of NAO. With the strengthening of the NAO signal, the winter monsoon developed a significant negative correlation, indicating that the strong 51–100-year NAO signal modulated the variation of winter monsoons. In our selected AO and AMO reconstructions, they were closely related to each other, with correlation coefficients of 0.34 at annual time scale and 0.89 at 100-year time scale. From Fig 5, the phase of the EAWM lagged by 15–20 years that of the AO and AMO variations, and if we moved the AO and AMO phases forward by 15–20 years, the correlation reached 0.88. That is, the variations of AO and AMO could be an early forecasting signal for the EAWM intensity.

It is worth noting that the PDO, AMO, AO and NAO modes play important roles in the modulation of EASM and EAWM; however, the East Asian monsoon system is complex and influenced by many internal and external climate forcings. The reasons for the EASM and EAWM variations at interannual—interdecadal—centennial time scales therefore need to be explored further by climate models.

## Conclusions

Based on observation data, we found that winter temperature and Meiyu precipitation in the MLRYR were highly correlated with the EAWM and EASM indices, respectively. Therefore, in this paper, the reconstructed winter temperature and Meiyu precipitation series from Chinese historical documents from 1736 to 2000 were used to represent long-term intensity variations in the EAWM and EASM. During the past 300 years, except for a coexisting 2–7-year interannual cycle, the EAWM had 31-year and 50–100-year oscillation signals and the EASM had 30–40-year and 100-year cycles, in which, the 50–100-year cycle of the EAWM negatively correlated with the NAO, and positively correlated with the AMO and AO, but with a 15–20-year time lag. The 30–40-year signal of the EASM had a positive correlation with the PDO.

In addition, we found that the phase relationships between the EAWM and EASM were not constant, and varied at different time scales. At interdecadal time scales (31-year), the EASM and EAWM were in phase. However, at centennial scales, the EASM and EAWM were out of phase, particularly before 1815; after then, a 20-year lagged summer monsoon phase existed. Although some main climatic modes at decadal time scales having close relationships with EAWM and EASM have been used for comparison, the influential mechanisms are complicated, particularly for the EASM, and the natural and internal forcings still require further investigations by model and data comparison.

## Supporting Information

S1 Table Original data for reconstructed Meiyu and winter temperature. (XLS)
